# Supplementary material for: The Influence of Storage Temperature and Packaging Technology on the Durability of Ready-to-Eat Preservative-Free Meat Bars with Dried Plasma
Source: Foods. 2023 Dec 4;12(23):4372. doi: 10.3390/foods12234372 (PMC10706313; doi:10.3390/foods12234372)
Supplement: Supplementary file 1 [file foods-12-04372-s001.zip › foods-2701104-supplementary.pdf]

Table S1. Mean log counts (with standard deviation) of APC, LAB, *S. aureus*, yeast and mould in VP_C and MAP_C RTE meat bars

Microorganisms tested	Storage time [= measurement days]	Packaging methods and storage temperatures				
		MAP		VP		
		4°C	8°C	4°C	6°C	8°C
APC	0	4.43^{aA}±0.14	4.43^{aA}±0.14	5.12^{aB}±0.16	5.12^{aB}±0.16	5.12^{aB}±0.16
	1	3.79^{aA}±0.59	4.12^{aA}±0.48	n/p	n/p	n/p
	3	3.68^{aA}±0.65	3.73^{aA}±0.57	5.23^{aB}±0.18	5.14^{aB}±0.19	5.39^{aB}±0.21
	7	4.40^{aA}±0.10	4.39^{aA}±0.40	5.00^{aB}±0.07	4.85^{aB}±0.10	5.04^{aB}±0.25
	10	4.17^{aA}±0.42	4.31^{aA}±0.76	n/p	n/p	n/p
	12	n/p	n/p	5.21^{aA}±0.49	5.29^{aA}±0.43	5.39^{aA}±0.14
	14	4.14^{aA}±0.21	4.16^{aA}±0.26	4.97^{aB}±0.01	5.44^{aB}±0.73	5.97^{aC}±0.16
	16	n/p	n/p	5.36^{aA}±0.16	5.03^{aA}±0.21	5.16^{aA}±0.28
	17	4.48^{aA}±0.19	5.21^{bB}±0.44	n/p	n/p	n/p
	21	4.35^{aA}±0.21	4.33^{aA}±0.27	5.30^{aB}±0.13	5.24^{aB}±0.02	5.00^{aB}±0.06
	24	5.14^{bA}±0.17	5.02^{bA}±0.24	n/p	n/p	n/p
	28	4.01^{aA}±0.33	4.41^{aA}±0.38	n/p	n/p	n/p
	35	3.95^{aA}±0.31	4.60^{aB}±0.54	n/p	n/p	n/p
LAB	0	2.02^{aA}±0.11	2.02^{aA}±0.11	3.76^{aB}±1.60	3.76^{aB}±1.60	3.76^{aB}±1.60
	1	2.02^{aA}±0.47	1.86^{aA}±0.43	n/p	n/p	n/p
	3	2.02^{aA}±0.23	2.06^{aA}±0.55	1.05^{bB}±0.07	1.35^{bB}±0.49	1.25^{bB}±0.36
	7	0.78^{bA}±0.25	0.67^{bA}±0.47	1.55^{bB}±0.41	1.52^{bB}±0.43	2.15^{cC}±0.81
	10	2.39^{aA}±0.32	2.47^{aA}±1.08	n/p	n/p	n/p
	12	n/p	n/p	2.92^{cA}±1.05	3.07^{aA}±1.54	3.83^{aB}±0.66
	14	1.37^{bA}±0.79	1.50^{cA}±0.41	2.19^{cB}±1.68	2.14^{cB}±0.53	2.23^{cB}±1.74
	16	n/p	n/p	2.73^{cA}±0.67	2.06^{cB}±0.96	2.45^{cA}±1.12
	17	2.75^{cA}±0.04	3.08^{dA}±0.43	n/p	n/p	n/p
	21	1.33^{bA}±0.47	2.79^{dB}±0.53	1.25^{bA}±0.36	1.32^{bA}±0.00	1.33^{bA}±0.46
	24	2.38^{aA}±0.32	3.80^{eB}±0.17	n/p	n/p	n/p
	28	1.95^{aA}±0.49	3.66^{eB}±0.36	n/p	n/p	n/p
	35	2.23^{aA}±0.69	3.94^{eB}±0.35	n/p	n/p	n/p
S. aureus	0	2.44^{aA}±0.39	2.44^{aA}±0.39	1.12^{aB}±0.79	1.12^{aB}±0.79	1.12^{aB}±0.79
	1	1.61^{bA}±0.48	1.48^{bA}±0.35	n/p	n/p	n/p
	3	1.02^{cA}±0.17	1.13^{bA}±0.11	1.58^{aA}±0.42	1.33^{aA}±0.46	1.39^{aA}±0.35
	7	2.06^{aA}±0.25	2.11^{aA}±0.26	1.00^{aB}±0.00	1.62^{aA}±0.67	1.11^{aB}±0.15
	10	0.77^{cA}±0.06	0.72^{cA}±0.18	n/p	n/p	n/p
	12	n/p	n/p	1.95^{bA}±0.67	1.78^{bA}±0.55	1.97^{bA}±0.74
	14	1.55^{bA}±0.17	2.65^{aB}±0.08	1.32^{aA}±0.45	2.20^{bB}±0.48	1.48^{aA}±0.34
	16	n/p	n/p	1.01^{aA}±0.83	1.32^{aA}±0.26	1.11^{aA}±0.15
	17	1.28^{cA}±0.28	1.36^{bA}±0.22	n/p	n/p	n/p
	21	2.38^{aA}±0.04	2.37^{aA}±0.13	2.00^{bA}±0.48	2.16^{bA}±0.61	2.16^{bA}±0.59
	24	1.84^{bA}±0.37	1.65^{bA}±0.32	n/p	n/p	n/p
	28	1.79^{bA}±0.56	1.25^{bB}±0.23	n/p	n/p	n/p
	35	1.73^{bA}±0.09	1.76^{bA}±0.07	n/p	n/p	n/p

Yeast and mould	0	0.00^{aA}±0.00	0.00^{aA}±0.00	0.33^{cB}±0.47	0.33^{bB}±0.47	0.33^{bB}±0.47
	1	0.00^{aA}±0.00	0.00^{aA}±0.00	n/p	n/p	n/p
	3	0.77^{bA}±1.09	0.00^{aB}±0.00	0.00^{aB}±0.00	0.00^{aB}±0.00	0.00^{aB}±0.00
	7	0.00^{aA}±0.00	0.00^{aA}±0.00	0.00^{aA}±0.00	0.00^{aA}±0.00	0.00^{aA}±0.00
	10	0.00^{aA}±0.00	0.00^{aA}±0.00	n/p	n/p	n/p
	12	n/p	n/p	1.16^{bA}±1.65	0.33^{bB}±0.47	0.33^{bB}±0.47
	14	0.00^{aA}±0.00	0.55^{bB}±0.77	0.62^{cB}±0.87	0.00^{aA}±0.00	1.52^{cC}±2.15
	16	n/p	n/p	0.00^{aA}±0.00	0.00^{aA}±0.00	0.00^{aA}±0.00
	17	0.83^{bA}±1.17	0.90^{bA}±1.27	n/p	n/p	n/p
	21	0.00^{aA}±0.00	0.00^{aA}±0.00	0.00^{aA}±0.00	0.00^{aA}±0.00	0.00^{aA}±0.00
	24	0.90^{bA}±1.27	0.90^{bA}±1.27	n/p	n/p	n/p
	28	0.00^{aA}±0.00	1.91^{cB}±0.40	n/p	n/p	n/p
	35	0.77^{bA}±0.56	0.00^{aB}±0.00	n/p	n/p	n/p

n/p – test was not performed on this measurement day
A, B, C – means in the same row marked with the same letters are not statistically different at α= 0.05
a, b, c, d, e - means in the same column marked with the same letters are not statistically different at α= 0.05
$p < 0.01$

Table S2. Mean log counts (with standard deviation) of APC, LAB, *S. aureus*, yeast and mould in VP_NC and MAP_NC RTE meat bars

**Microorganisms tested**	**Storage time [= measurement day]**	**Packaging methods and storage temperature**									
		**MAP**						**VP**			
		**12 °C**	**16 °C**	**20 °C**	**24 °C**	**30 °C**	**34 °C**	**12 °C**	**16 °C**	**20 °C**	**24 °C**
APC	0	4.43^{aA}±0.14	4.43^{aA}±0.14	4.43^{aA}±0.14	4.43^{aA}±0.14	4.43^{aA}±0.14	4.43^{aA}±0.14	5.12^{aB}±0.16	5.12^{aB}±0.16	5.12^{aB}±0.16	5.12^{aB}±0.16
	1	3.93^{aA}±0.62	4.09^{aA}±0.32	4.50^{aB}±0.15	4.99^{bB}±0.26	5.99^{bC}±0.34	5.28^{bC}±0.55	n/p	n/p	n/p	n/p
	3	4.47^{aA}±0.22	5.15^{aB}±0.59	4.79^{aB}±0.66	6.29^{cC}±0.38	7.27^{cD}±0.67	5.93^{cC}±0.65	5.07^{aB}±0.23	5.34^{aB}±0.19	5.06^{aB}±0.46	5.40^{aB}±0.82
	7	4.34^{aA}±0.16	5.01^{aB}±0.88	5.60^{bB}±0.88	6.94^{dC}±0.18	7.07^{cC}±0.54	7.26^{dC}±0.50	5.48^{aB}±0.41	5.49^{aB}±0.69	5.42^{aB}±0.87	5.84^{aB}±0.98
	10	4.57^{aA}±0.19	5.55^{bB}±0.76	6.61^{cC}±1.06	7.83^{eD}±0.67	8.84^{dE}±0.42	8.85^{eE}±0.10	n/p	n/p	n/p	n/p
	12	n/p	n/p	n/p	n/p	n/p	n/p	5.11^{aA}±0.30	5.29^{aA}±0.38	5.62^{aB}±0.65	5.78^{aB}±0.64
	14	4.77^{aA}±0.67	5.20^{bA}±1.06	6.83^{cB}±0.71	8.02^{eC}±0.29	8.08^{eC}±0.28	7.15^{dB}±0.39	5.45^{aD}±0.18	4.89^{aA}±0.18	4.86^{aA}±0.08	5.61^{bD}±0.16
	16	n/p	n/p	n/p	n/p	n/p	n/p	5.58^{aA}±0.70	5.50^{aA}±0.76	5.46^{aA}±0.66	5.15^{aA}±0.98
	17	6.40^{bA}±0.42	6.96^{cB}±0.17	7.16^{cC}±0.26	7.81^{eD}±0.08	8.09^{eD}±0.28	7.69^{dD}±0.39	n/p	n/p	n/p	n/p
	21	6.03^{bA}±0.71	6.55^{cB}±0.57	7.43^{cC}±0.28	8.29^{eD}±0.30	8.57^{dD}±0.36	6.59^{dB}±0.27	5.04^{aE}±0.06	5.06^{aE}±0.16	5.09^{aE}±0.15	4.93^{aE}±0.06
	24	5.96^{bA}±0.45	5.75^{bA}±0.86	7.53^{cB}±0.64	8.05^{eC}±0.11	8.57^{dC}±0.61	7.45^{dB}±0.32	n/p	n/p	n/p	n/p
	28	6.34^{bA}±0.46	6.80^{cB}±0.19	7.65^{cC}±0.06	8.01^{eD}±0.27	8.22^{eD}±0.59	6.40^{cA}±0.43	n/p	n/p	n/p	n/p
	35	6.38^{bA}±0.51	7.08^{cB}±0.22	7.94^{dC}±0.20	8.01^{eC}±0.17	7.49^{cD}±0.12	6.88^{dB}±0.17	n/p	n/p	n/p	n/p
LAB	0	2.02^{aA}±0.11	2.02^{aA}±0.11	2.02^{aA}±0.11	2.02^{aA}±0.11	2.02^{aA}±0.11	2.02^{aA}±0.11	3.76^{aA}±1.60	3.76^{aA}±1.60	3.76^{aA}±1.60	3.76^{aA}±1.60
	1	1.86^{aA}±0.68	2.04^{aA}±0.50	3.76^{bB}±0.46	3.57^{bB}±0.30	4.99^{bC}±0.51	4.94^{bC}±0.67	n/p	n/p	n/p	n/p
	3	2.37^{aA}±0.62	4.21^{bB}±0.35	4.21^{bB}±0.54	4.92^{cC}±0.17	6.06^{cD}±0.11	5.75^{cD}±0.72	1.73^{bA}±1.04	1.69^{bA}±0.98	1.33^{bA}±0.47	1.56^{bA}±0.79
	7	3.45^{bA}±0.67	4.07^{bA}±0.64	4.76^{cB}±0.31	5.29^{cB}±0.57	6.53^{cC}±0.55	7.98^{dD}±0.09	2.8^{cA}±1.43	2.41^{cA}±1.79	2.17^{cA}±1.31	3.24^{aA}±1.23
	10	4.65^{cA}±0.76	4.50^{bA}±0.91	5.37^{cA}±1.05	7.14^{dB}±0.40	6.86^{cB}±0.74	7.73^{dC}±0.13	n/p	n/p	n/p	n/p

	12	n/p	n/p	n/p	n/p	n/p	n/p	3.33^{aA}±1.66	2.07^{cA}±1.29	3.72^{aA}±2.00	3.15^{aA}±0.94
	14	4.12^{cA}±0.47	5.12^{cB}±0.36	6.02^{dC}±0.27	6.76^{dD}±0.27	7.53^{dE}±0.37	7.98^{dF}±0.15	2.58^{cG}±1.50	1.55^{bH}±0.77	3.34^{aG}±0.00	3.22^{cG}±0.25
	16	n/p	n/p	n/p	n/p	n/p	n/p	3.26^{aA}±1.34	3.51^{aA}±1.10	4.10^{aA}±0.75	4.28^{aA}±0.40
	17	5.30^{dA}±0.84	5.97^{dA}±0.54	6.90^{eB}±0.24	7.50^{dC}±0.54	8.03^{dC}±0.24	7.88^{dC}±0.32	n/p	n/p	n/p	n/p
	21	4.86^{dA}±0.47	5.95^{dB}±0.44	6.84^{eC}±0.37	7.64^{dD}±0.77	8.29^{dE}±0.42	7.13^{eC}±0.33	1.00^{bF}±0.00	1.32^{bF}±0.00	1.67^{bF}±0.25	1.11^{aF}±0.15
	24	5.04^{dA}±0.38	5.23^{cA}±0.35	6.16^{dB}±0.42	6.73^{dB}±0.72	8.70^{eC}±0.09	6.76^{eB}±0.41	n/p	n/p	n/p	n/p
	28	5.06^{dA}±0.44	6.31^{dB}±0.79	6.40^{dB}±0.51	7.55^{dC}±0.50	7.79^{dC}±0.09	6.87^{eB}±0.78	n/p	n/p	n/p	n/p
	35	5.43^{dA}±0.48	6.73^{dB}±0.68	5.86^{dA}±0.20	6.04^{cB}±0.69	7.31^{dC}±0.29	6.55^{eB}±0.35	n/p	n/p	n/p	n/p
S. aureus	0	2.44^{aA}±0.39	2.44^{aA}±0.39	2.44^{aA}±0.39	2.44^{aA}±0.39	2.44^{aA}±0.39	2.44^{aA}±0.39	1.12^{aB}±0.79	1.12^{aB}±0.79	1.12^{aB}±0.79	1.12^{aB}±0.79
	1	1.15^{bA}±0.16	1.26^{bA}±0.37	1.18^{bA}±0.33	1.73^{bA}±0.25	1.82^{bA}±0.21	1.91^{bB}±0.16	n/p	n/p	n/p	n/p
	3	1.23^{bA}±0.22	1.52^{bA}±0.39	3.47^{cB}±0.29	1.59^{bA}±0.47	3.84^{cB}±0.11	3.79^{cB}±0.06	1.00^{aA}±0.00	1.28^{aA}±0.21	1.00^{aA}±0.00	1.33^{aA}±0.47
	7	1.93^{cA}±0.28	2.32^{aB}±0.22	2.37^{aB}±0.22	1.77^{bA}±0.33	0.00^{dC}±0.00	2.41^{aB}±0.78	1.30^{aD}±0.25	1.00^{aD}±0.00	1.22^{aD}±0.31	1.44^{aD}±0.62
	10	2.21^{aA}±0.23	1.81^{cA}±0.69	3.23^{cB}±0.32	3.31^{cB}±0.76	3.08^{eB}±0.28	3.51^{cB}±0.33	n/p	n/p	n/p	n/p
	12	n/p	n/p	n/p	n/p	n/p	n/p	2.02^{bA}±1.02	2.01^{bA}±0.75	2.18^{bA}±0.91	1.72^{aA}±0.69
	14	1.97^{aA}±0.33	1.82^{cA}±0.14	2.17^{aA}±0.41	1.96^{bA}±0.24	2.16^{aA}±0.18	1.92^{bA}±0.69	2.19^{bA}±0.61	1.17^{aB}±0.24	1.00^{aB}±0.00	1.00^{aB}±0.00
	16	n/p	n/p	n/p	n/p	n/p	n/p	1.00^{aA}±0.00	1.11^{aA}±0.15	1.21^{aA}±0.30	1.15^{aA}±0.22
	17	0.96^{bA}±0.15	1.82^{cB}±0.14	2.37^{aC}±0.34	1.83^{bB}±0.21	4.06^{cD}±0.34	3.17^{dE}±0.16	n/p	n/p	n/p	n/p
	21	1.44^{bA}±0.70	1.74^{bA}±0.68	1.48^{bA}±0.59	2.24^{aB}±0.46	0.91^{fC}±0.71	1.40^{bA}±0.67	1.21^{aA}±0.15	1.80^{bA}±0.57	1.00^{aC}±0.00	1.74^{aA}±0.52
	24	1,84^{aA}±0.46	1,65^{bA}±0.67	1,56^{bA}±0.58	1,59^{bA}±0.53	1,49^{bA}±0.66	1,76^{bA}±0.53	n/p	n/p	n/p	n/p
	28	1.25^{bA}±0.23	1.66^{bB}±0.48	1.15^{bA}±0.35	1.16^{bA}±1.02	1.83^{bB}±0.42	1.36^{bA}±0.39	n/p	n/p	n/p	n/p
	35	1.79^{aA}±0.56	1.45^{bA}±0.60	0.92^{bB}±0.02	1.36^{bA}±0.97	1.94^{bA}±0.24	1.18^{bA}±0.28	n/p	n/p	n/p	n/p
Yeast and mould	0	0.00^{aA}±0.00	0.00^{aA}±0.00	0.00^{aA}±0.00	0.00^{aA}±0.00	0.00^{aA}±0.00	0.00^{aA}±0.00	0.33^{aB}±0.47	0.33^{aB}±0.47	0.33^{aB}±0.47	0.33^{aB}±0.47
	1	0.00^{aA}±0.00	0.00^{aA}±0.00	0.00^{aA}±0.00	2.52^{bB}±0.63	0.58^{bC}±0.82	0.00^{aA}±0.00	n/p	n/p	n/p	n/p
	3	0.00^{aA}±0.00	2.34^{bB}±1.66	2.16^{bB}±1.59	2.86^{bB}±0.62	3.01^{cB}±2.14	4.41^{bC}±0.33	0.00^{bA}±0.00	0.00^{bA}±0.00	0.00^{bA}±0.00	0.00^{bA}±0.00
	7	0.00^{aA}±0.00	0.74^{cB}±0.53	1.17^{cB}±0.24	0.88^{cB}±0.68	2.99^{cC}±1.39	2.32^{cC}±0.78	0.00^{bA}±0.00	0.00^{bA}±0.00	0.00^{bA}±0.00	0.00^{bA}±0.00
	10	1.00^{bA}±0.73	3.78^{dB}±0.66	2.58^{bC}±1.16	2.90^{bC}±2.07	3.67^{dB}±1.71	4.48^{bD}±0.40	n/p	n/p	n/p	n/p
	12	n/p	n/p	n/p	n/p	n/p	n/p	0.00^{bA}±0.00	0.00^{bA}±0.00	1.55^{cB}±1.28	0.00^{bA}±0.00
	14	1.01^{bA}±1.43	2.23^{bB}±1.92	2.23^{bB}±1.11	4.68^{dC}±0.01	5.42^{eD}±0.42	2.55^{cB}±0.37	1.26^{cA}±1.78	0.00^{bE}±0.00	0.00^{aE}±0.00	0.00^{bE}±0.00
	16	n/p	n/p	n/p	n/p	n/p	n/p	0.00^{bA}±0.00	0.00^{bA}±0.00	1.22^{cB}±1.73	0.00^{bA}±0.00
	17	2.83^{cA}±0.95	4.69^{eB}±0.22	3.46^{dC}±1.74	3.10^{bA}±2.19	5.88^{fD}±0.15	6.03^{dD}±0.31	n/p	n/p	n/p	n/p
	21	2.99^{cA}±1.05	3.05^{fA}±1.26	3.07^{dA}±0.90	4.15^{dB}±1.33	3.44^{eC}±1.11	3.53^{eC}±0.59	0.00^{bD}±0.00	0.00^{bD}±0.00	1.47^{cE}±2.07	0.00^{bD}±0.00
	24	3.69^{dA}±1.13	4.72^{eB}±0.13	3.90^{eA}±1.35	3.27^{bA}±2.32	5.86^{fC}±0.15	4.48^{bB}±0.58	n/p	n/p	n/p	n/p
	28	0.99^{bA}±1.40	4.45^{eB}±0.22	2.21^{bC}±0.09	4.60^{dB}±1.09	4.33^{gB}±0.18	1.50^{fA}±0.50	n/p	n/p	n/p	n/p
	35	2.39^{cA}±1.69	3.37^{dB}±1.44	2.84^{dC}±1.51	3.23^{bB}±1.03	2.49^{cA}±0.41	1.00^{fD}±0.82	n/p	n/p	n/p	n/p

n/p – test was not performed on this measurement day

A, B, C, D, E, F, G, H – means in the same row marked with the same letters are not statistically different at α= 0.05

a, b, c, d, e, f - means in the same column marked with the same letters are not statistically different at α= 0.05

$p < 0.01$

Table S3. Mean values of pH and w_a of RTE meat bars during storage (MAP_C, VP_C, MAP_NC, VP_NC)

Temperature [°C]	Packaging technique	Factor	Storage time [day]								
			0	3	7	12	14	16	21	28	35
4	VP	pH	6.08	6.12	6.05	6.09	5.94	5.95	6.07	n/p	n/p
		Wa	0.945	0.947	0.948	0.951	0.944	0.946	0.947	n/p	n/p
	MAP	pH	6.06	n/p	6.09	n/p	6.10	n/p	6.10	6.08	6.13
		Wa	0.953	n/p	0.942	n/p	0.943	n/p	0.952	0.952	0.947
6	VP	pH	6.08	6.10	6.04	6.09	5.95	5.96	6.07	n/p	n/p
		Wa	0.945	0.945	0.948	0.945	0.943	0.948	0.943	n/p	n/p
8	VP	pH	6.08	6.12	6.05	6.10	5.98	5.96	6.05	n/p	n/p
		Wa	0.945	0.946	0.949	0.944	0.945	0.947	0.944	n/p	n/p
	MAP	pH	6.06	n/p	6.09	n/p	6.10	n/p	6.10	6.09	6.12
		Wa	0.953	n/p	0.951	n/p	0.946	n/p	0.947	0.948	0.947
12	VP	pH	6.08	6.12	6.04	6.11	5.98	5.97	6.08	n/p	n/p
		Wa	0.945	0.943	0.950	0.946	0.940	0.946	0.942	n/p	n/p
	MAP	pH	6.06	n/p	6.10	n/p	6.09	n/p	6.09	6.08	6.11
		Wa	0.953	n/p	0.947	n/p	0.946	n/p	0.947	0.948	0.945
16	VP	pH	6.08	6.07	6.03	6.12	5.98	5.97	6.05	n/p	n/p
		Wa	0.945	0.942	0.949	0.944	0.941	0.948	0.945	n/p	n/p
	MAP	pH	6.06	n/p	6.10	n/p	6.06	n/p	6.09	6.06	6.09
		Wa	0.953	n/p	0.948	n/p	0.951	n/p	0.948	0.949	0.946
20	VP	pH	6.08	6.08	6.04	6.10	5.98	6.00	6.07	n/p	n/p
		Wa	0.945	0.940	0.945	0.944	0.939	0.946	0.947	n/p	n/p
	MAP	pH	6.06	n/p	6.09	n/p	5.92	n/p	5.78	5.68	6.03
		Wa	0.953	n/p	0.947	n/p	0.948	n/p	0.947	0.948	0.946
24	VP	pH	6.08	6.09	6.04	6.12	5.97	5.97	6.06	n/p	n/p
		Wa	0.945	0.943	0.949	0.951	0.946	0.943	0.943	n/p	n/p
	MAP	pH	6.06	n/p	6.07	n/p	5.88	n/p	5.67	5.51	5.98
		Wa	0.953	n/p	0.946	n/p	0.944	n/p	0.942	0.939	0.939
30	VP	pH	6.06	n/p	6.05	n/p	5.66	n/p	5.71	5.37	5.38
		Wa	0.953	n/p	0.947	n/p	0.939	n/p	0.936	0.923	0.916
34	MAP	pH	6.06	n/p	5.88	n/p	5.48	n/p	5.50	5.23	5.45
		Wa	0.953	n/p	0.945	n/p	0.924	n/p	0.920	0.908	0.904

n/p – test was not performed on this measurement day
